# Supplementary material for: Confounding Factors Influencing the Kinetics and Magnitude of Serological Response Following Administration of BNT162b2
Source: Microorganisms. 2021 Jun 21;9(6):1340. doi: 10.3390/microorganisms9061340 (PMC8235462; doi:10.3390/microorganisms9061340)
Supplement: Supplementary file 1 [file microorganisms-09-01340-s001.zip › microorganisms-1271374-supplementary.pdf]

Table S1. Multivariable linear regression for Anti-S/RBD IgG antibody titers**.** Statistically significant variables are marked in bold for each (sub) analyses performed. The regression coefficient (i.e the β estimate) obtained shows the increase or decrease in log_{10}-transformed anti-Spike/RBD values outcome per unit increase in the factor studied, adjusted for the effect of other variables in the model. Results reported the β estimate, its 95% confidence interval, and the corresponding p-value for the variable of interest in a particular model. The adjusted R-square is also reported for each model run. R-square for multicollinearity was always below 0.20 for all model tested.

	Day 14		Day 28		Day 42	
**Variable**	**Beta (95%CI)**	**p-value**	**Beta (95%CI)**	**p-value**	**Beta (95%CI)**	**p-value**
**Full cohort**	**N analyzed = 192, Adjusted R^2: 0.778**		**N analyzed = 196, Adjusted R^2: 0.115**		**N analyzed = 184, Adjusted R^2: 0.103**	
Sex (male)	-0.09812 (-0.2448 to 0.04860)	0.1887	0.07499 (-0.2018 to 0.09401)	0.4730	-0.0981 (-0.202 to 0.00636)	0.0655
**Age (years)**	**-0.01259 (-0.01800 to -0.007172)**	**<0.0001**	**-0.002714 (-0.01145 to -0.0007421)**	**0.0259**	-0.00336 (-0.00712 to 0.000400)	0.0795
BMI (kg/m2)	0.009489 (-0.005105 to 0.02408)	0.2012	0.007403 (-0.007995 to 0.02121)	0.3732	0.00354 (-0.00676 to 0.0138)	0.4985
Blood group A	0.02738 (-0.1103 to 0.1651)	0.6953	0.06961 (-0.1417 to 0.1329)	0.9494	-0.0209 (-0.116 to 0.0739)	0.6640
**Blood group AB**	**-0.3152 (-0.6225 to -0.007893)**	**0.0445**	0.1577 (-0.3702 to 0.2520)	0.7083	-0.138 (-0.374 to 0.0975)	0.2489
Blood group B	0.1721 (-0.05609 to 0.4002)	0.1385	0.1167 (-0.2422 to 0.2182)	0.9184	0.0249 (-0.149 to 0.199)	0.7779
**Previous SARS-CoV-2**	**1.683 (1.545 to 1.821)**	**<0.0001**	**0.06957 (0.1473 to 0.4218)**	**<0.0001**	**0.209 (0.111 to 0.307)**	**<0.0001**
**SARS-CoV-2 Naïve**	**N analyzed = 133, Adjusted R^2: 0.160**		**N analyzed = 134, Adjusted R^2: 0.044**		**N analyzed = 131, Adjusted R^2: 0.070**	
**Sex (male)**	-0.153 (-0.3281 to 0.02204)	0.0861	-0.07752 (-0.2753 to 0.1202)	0.4393	**-0.1286 (-0.2509 to -0.006370)**	**0.0394**
**Age (years)**	**-0.01474 (-0.02135 to -0.008119)**	**<0.0001**	**-0.007837 (-0.01524 to -0.0004362)**	**0.0381**	-0.00413 (-0.008652 to 0.0003919)	0.0731
BMI (kg/m^2)	0.007087 (-0.01012 to 0.02429)	0.4165	0.005132 (-0.01392 to 0.02418)	0.5949	0.000847 (-0.01085 to 0.01254)	0.8863
Blood group (A)	0.002837 (-0.1647 to 0.1703)	0.9733	-0.02639 (-0.2117 to 0.1589)	0.7785	-0.03809 (-0.1517 to 0.07554)	0.5083
Blood group (AB)	-0.1533 (-0.5041 to 0.1975)	0.3888	-0.01729 (-0.4108 to 0.3762)	0.9308	-0.1421 (-0.3979 to 0.1136)	0.2734
Blood group (B)	0.1333 (-0.1451 to 0.4117)	0.3453	-0.1138 (-0.4253 to 0.1976)	0.4709	0.0408 (-0.1654 to 0.2470)	0.6960
**Previous SARS-CoV-2**	**N analyzed = 59, Adjusted R^2: 0.294**		**N analyzed = 62, Adjusted R^2: 0.114**		**N analyzed = 53, Adjusted R^2: 0.056**	
Sex (male)	0.06036 (-0.2002 to 0.3209)	0.6440	-0.003949 (-0.2018 to 0.1939)	0.9682	-0.009967 (-0.2211 to 0.2012)	0.09502
Age (years)	-0.002444 (-0.01219 to 0.007299)	0.6168	-0.0008248 (-0.007887 to 0.006237)	0.8158	-0.0008263 (-0.008100 to 0.006448)	0.2286
BMI (kg/m^2)	0.01918 (-0.007521 to 0.04589)	0.1554	0.01466 (-0.006176 to 0.03549)	0.1642	0.01646 (-0.007005 to 0.03992)	1.412
Blood group (A)	0.1692 (-0.07606 to 0.4145)	0.1721	0.09159 (-0.09532 to 0.2785)	0.3304	0.06968 (-0.1200 to 0.2594)	0.7393
Blood group (AB)	0.2079 (-0.1760 to 0.5917)	0.2822	0.1837 (-0.1153 to 0.4827)	0.2235	-0.0323 (-0.3751 to 0.3105)	0.1897
**Blood group (B)**	**-1.086 (-1.713 to -0.4597)**	**0.0010**	-0.3325 (-0.8222 to 0.1572)	0.1792	-0.1932 (-0.8326 to 0.4462)	0.6083
**Female gender**	**N analyzed = 143, Adjusted R^2: 0.759**		**N analyzed = 147, Adjusted R^2: 0.130**		**N analyzed = 140, Adjusted R^2: 0.107**	
**Age (years)**	**-0.01167 (-0.02280 to -0.0005468)**	**0.0399**	-0.001849 (-0.01232 to 0.008620)	0.7275	-0.003665 (-0.01108 to 0.003752)	0.3302
BMI (kg/m^2)	0.01109 (-0.005272 to 0.02745)	0.1824	0.01051 (-0.005007 to 0.02603)	0.1827	0.005487 (-0.005657 to 0.01663)	0.3318
Blood group A	0.0541 (-0.1096 to 0.2178)	0.5146	-0.01694 (-0.1718 to 0.1379)	0.8291	-0.03186 (-0.1408 to 0.07710)	0.5639
**Blood group AB**	**-0.4756 (-0.8663 to -0.08498)**	**0.0174**	-0.06347 (-0.4398 to 0.3129)	0.7393	-0.2011 (-0.4609 to 0.05870)	0.1281
Blood group B	0.1385 (-0.1621 to 0.4390)	0.3638	0.1689 (-0.1196 to 0.4575)	0.2491	-0.002485 (-0.2374 to 0.2325)	0.9833
Menopaused	-0.08219 (-0.3504 to 0.1860)	0.5455	-0.1477 (-0.4021 to 0.1067)	0.2528	0.04522 (-0.1385 to 0.2290)	0.6272
**Previous SARS-CoV-2**	**1.625 (1.457 to 1.792)**	**<0.0001**	**0.2627 (0.1052 to 0.4203)**	**0.0012**	**0.1854 (0.07177 to 0.2990)**	**0.0016**
**Childbearing age**	**N analyzed = 98, Adjusted R^2: 0.831**		**N analyzed = 100, Adjusted R^2: 0.169**		**N analyzed = 96, Adjusted R^2: 0.194**	
**Age (years)**	**-0.01433 (-0.02458 to -0.004071)**	**0.0067**	-0.001916 (-0.01127 to 0.007436)	0.6850	-0.00417 (-0.01114 to 0.002796)	0.2374
**BMI (kg/m^2)**	**0.02571 (0.006468 to 0.04495)**	**0.0094**	0.01571 (-0.001809 to 0.03323)	0.0782	0.01136 (-0.001957 to 0.02468)	0.0936

Blood group A	-0.003061 (-0.1658 to 0.1597)	0.9703	0.01863 (-0.1309 to 0.1682)	0.8051	0.0009382 (-0.1094 to 0.1112)	0.9866
**Blood group AB**	**-0.5378 (-0.8907 to -0.1849)**	**0.0032**	-0.01882 (-0.3476 to 0.3100)	0.9097	-0.1414 (-0.3783 to 0.09552)	0.2388
Blood group B	0.02588 (-0.2813 to 0.3331)	0.8674	0.1086 (-0.1742 to 0.3914)	0.4477	-0.1106 (-0.3314 to 0.1102)	0.3222
Use of contraceptive	-0.03245 (-0.1964 to 0.1315)	0.6951	0.07693 (-0.07597 to 0.2298)	0.3203	0.07073 (-0.04599 to 0.1874)	0.2317
**Previous SARS-CoV-2**	**1.604 (1.445 to 1.763)**	**<0.0001**	**0.2596 (0.1122 to 0.4070)**	**0.0007**	**0.1964 (0.08644 to 0.3063)**	**0.0006**
**Menopausal**	**N analyzed = 40, Adjusted R^2: 0.059**		**N analyzed = 42, Adjusted R^2: 0.089**		**N analyzed = 39, Adjusted R^2: 0.148**	
Age (years)	-0.0004572 (-0.04205 to 0.04113)	0.9823	-0.01397 (-0.05149 to 0.02355)	0.4546	-0.00341 (-0.02931 to 0.02249)	0.7901
BMI (kg/m^2)	-0.008783 (-0.04905 to 0.03148)	0.6598	0.006668 (-0.03101 to 0.04435)	0.7213	0.003677 (-0.02220 to 0.02955)	0.7739
Blood group A	0.2474 (-0.2391 to 0.7338)	0.3080	-0.09809 (-0.5421 to 0.3459)	0.6563	-0.11 (-0.4161 to 0.1961)	0.4692
Blood group B	0.04948 (-0.8481 to 0.9470)	0.9113	0.0671 (-0.7753 to 0.9095)	0.8724	0.5643 (-0.3416 to 1.470)	0.2134
Blood group AB	-0.5564 (-2.042 to 0.9292)	0.4511	-0.472 (-1.865 to 0.9214)	0.4959	-0.6349 (-1.596 to 0.3258)	0.1875
Use of HRT	0.2958 (-0.2526 to 0.8441)	0.2801	0.2435 (-0.2575 to 0.7445)	0.3302	0.1494 (-0.2129 to 0.5116)	0.4068
**Previous SARS-CoV-2**	**1.54 (1.001 to 2.078)**	**<0.0001**	0.1739 (-0.3010 to 0.6487)	0.4619	0.1337 (-0.2194 to 0.4867)	0.4458

Abbreviations: BMI, body mass index; CI, confidence interval; HRT, hormone replacement therapy; R^2, R-square.
